# Change of the child’s posture after sacroiliac joint manipulation: improved symmetry assessed withthe POTSI index

**DOI:** 10.1186/1748-7161-7-S1-O65

**Published:** 2012-01-27

**Authors:** L Stolinski, T Kotwicki

**Affiliations:** 1 Rehasport Clinic, Poznan, Poland; Sports Secondary School Complex the John Paul II, Skierniewice, Poland; 2 Spine Disorders Unit Department of Pediatric Orthopedics and Traumatology, University of Medical Sciences, Poznan, Poland; 3 Sports Secondary School Complex the John Paul II, Skierniewice, Poland

## Purpose of the study

To investigate the influence of a single procedure of manipulation of the sacroiliac joints according to Ackermann on the posture of the child, assessed with digital photographs using Posterior Trunk Symmetry Index (POTSI) [1-4].

## Background

The use of joint mobilization and manipulation in pediatric patients is a controversial topic due to lack of data respecting Evidence Based Medicine.

## Materials and methods

The study group comprised 39 children (17 girls, 22 boys), aged 7.0 to 11.0, mean 8.8 ± 1.1, having the “twisted pelvis” defined as a combination of nutation of one iliac bone and contra-nutation of another iliac bone as well as an apparent shortness of one leg in supine position. The control group comprised 39 children (22 girls, 17 boys), aged 7.0 to 11.0, mean 9.0 ± 1.4. The groups were matched for age, height, weight and BMI. Digital photos of the trunk in standing habitual posture were performed twice: before and after manual therapy comprising single manipulation of the sacroiliac joints according to Ackermann. The control group had no therapy but just a 5-minute rest in sitting position between the two photos.

## Results

In the study group POTSI improved significantly from 26.1 ± 12.0 to 16.8 ± 9.5. In the control group POTSI did not change: 21.7 ± 10.3 versus 21.3 ± 11.1.

## Conclusions

Single mobilization of the sacroiliac joints by Ackermann method allows for improvement of posture symmetry in children. Photographic assessment of posture using the POTSI index can be used to document it.

## References

[B1] Ackermann WP Die gezielte Diagnose und Technik der Chiropraktik 2008 USP Publishing

[B2] Mínguez M Buendía M Cibrián R Salvador R Laguía M Martín A Gomar F Quantifier variables of the back surface deformity obtained with a noninvasive structured light method: evaluation of their usefulness in idiopathic scoliosis diagnosis Eur Spine J 2007 16 73 82 10.1007/s00586-006-0079-y 16609858 PMC2198893

[B3] Suzuki N Inami K Ono T Kohno K Asher MA Analysis of posterior trunk symmetry index (POTSI) in scoliosis, part 1 Stud Health Technol Inform 1999 59 81 84

[B4] Inami K Suzuki N Ono T Yamashita Y Kohno K Morisue H Analysis of posterior trunk symmetry index (POTSI) in scoliosis, part 2 Stud Health Technol Inform 1999 59 85 88

